# Shifting the Paradigm: A Quality Improvement Approach to Proactive Cardiac Arrest Reduction in the Pediatric Cardiac Intensive Care Unit

**DOI:** 10.1097/pq9.0000000000000525

**Published:** 2022-01-21

**Authors:** Christine M. Riley, J. Wesley Diddle, Ashleigh Harlow, Kara Klem, Jason Patregnani, Evan Hochberg, Jenhao Jacob Cheng, Sopnil Bhattarai, Lisa Hom, Justine M. Fortkiewicz, Darren Klugman

**Affiliations:** From the * Children’s National Hospital, Division of Cardiac Critical Care Medicine, Washington, D.C.; † Division of Nursing, Children’s National Hospital, Cardiac Intensive Care Unit, Washington, D.C.; ‡ Cardiac Acute and Critical Care, UPMC Children’s Hospital of Pittsburgh, Pittsburgh, Pa.; § Division of Pediatric Critical Care, Barbara Bush Children’s Hospital at Maine Medical Center, Portland, Maine; ¶ Business Process Consultant, University of Maryland-College Park, College Park, Md.; ∥ Division of Quality and Patient Safety, Children’s National Hospital, Washington, D.C.; ** Division of Safety and Performance Improvement, Children’s National Hospital, Washington, D.C.; †† Heart Center, Children’s National Hospital, Washington, D.C.; ‡‡ Division of Anesthesia Critical Care Medicine, Johns Hopkins Children’s Center.

## Abstract

**Introduction:**

Children with cardiac conditions are at higher risk of in-hospital pediatric cardiopulmonary arrest (CA), resulting in significant morbidity and mortality. Despite the elevated risk, proactive cardiac arrest prevention programs in the cardiac intensive care unit (CICU) remain underdeveloped. Our team developed a multidisciplinary program centered on developing a quality improvement (QI) bundle for patients at high risk of CA.

**Methods:**

This project occurred in a 26-bed pediatric CICU of a tertiary care children’s hospital. Statistical process control methodology tracked changes in CA rates over time. The global aim was to reduce CICU mortality; the smart aim was to reduce the CA rate by 50% over 12 months. Interprofessional development and implementation of a QI bundle included visual cues to identify high-risk patients, risk mitigation strategies, a new rounding paradigm, and defined escalation algorithms. Additionally, weekly event and long-term data reviews, arrest debriefs, and weekly unit-wide dissemination of key findings supported a culture change.

**Results:**

After bundle implementation, CA rates decreased by 68% compared to baseline and 45% from the historical baseline. Major complications decreased from 17.1% to 12.6% (*P* < 0.001) and mortality decreased from 5.7% to 5.0% (*P* = 0.048). These results were sustained for 30 months.

**Conclusions:**

Cardiac arrest is a modifiable, rather than inevitable, metric in the CICU. Reduction is achievable through the interprofessional implementation of bundled interventions targeting proactive CA prevention. Once incorporated into widespread efforts to engage multidisciplinary CICU stakeholders, these patient-focused interventions resulted in sustained improvement.

## Abstract 2

Supplemental Digital Content is available in the text.

## INTRODUCTION

Approximately 15,200 children experience in-hospital cardiac arrest every year.^1^ Children with underlying cardiac conditions experience higher rates of cardiopulmonary arrest (CA) than the general pediatric intensive care population^2,3^ resulting in significant morbidity and mortality.^2,4,5^ Although recent multicenter data have shown that 78% of children who experience an in-hospital CA in an intensive care unit achieve a return of circulation, only 45% survive to discharge, and postarrest morbidity is substantial.^6,7^ Despite advances in team performance and care delivery,^8–11^ Cardiac intensive care unit (CICU) patients remain at risk for adverse events, including CA. Thus, CA may represent a failure to match resources and assess the degree of patient risk adequately.

Unit- and patient-level factors catalyzed a paradigm shift in our approach to CA, prompting a focus on proactive cardiac arrest prevention (CAP). There was a notable increase in the CA rate at the unit level from late 2015 to early 2016. At the patient level, a child with high-risk physiology, under the care of some of our most experienced medical and nursing staff, had a witnessed-postoperative CA. Unfortunately, the family faced a devastating neurological outcome despite receiving prompt, high-quality cardiopulmonary resuscitation (CPR) and rapid extracorporeal CPR (ECPR) cannulation. The formal review identified no obvious opportunities to improve the resuscitation response, so subsequent efforts focused on proactive arrest prevention.

Advances in resuscitation science and performance have demonstrated several significant findings that improve outcomes following CA.^12–19^ Improving the quality with which teams deliver CPR is essential and may prevent CA from leading to mortality.^20^ However, improvement work focusing on the quality of CPR during and care after an arrest differs conceptually from that of proactive CAP. Few manuscripts have described pediatric CA rates in the intensive care setting as a modifiable metric or successfully implemented proactive CA reduction strategies with a documented sustained decrease. Ferguson et al^21^ reported a single targeted intervention of placing prepared code-dose epinephrine at the bedside of high-risk patients, which was associated with a decreased rate of CA in pediatric cardiac patients.^21^ Review of adult studies suggested that intervention in the period before CA and proactive engagement of frontline staff are required to prevent CA.^22,23^

The global aim of this QI project was to reduce CICU mortality, and the smart aim was to reduce the CA rate by 50% over 12 months and sustain that decrease for six months.

## METHODS

The setting for this study was an academic, tertiary care pediatric center with 316 beds. The pediatric cardiac surgical program performs more than 300 index cases per year, and the 26-bed CICU has approximately 750 admissions per year. The Children’s National Institutional Review Board reviewed this quality improvement (QI) project and deemed it exempt (approval number: Pro00009161).

**Fig. 1.**
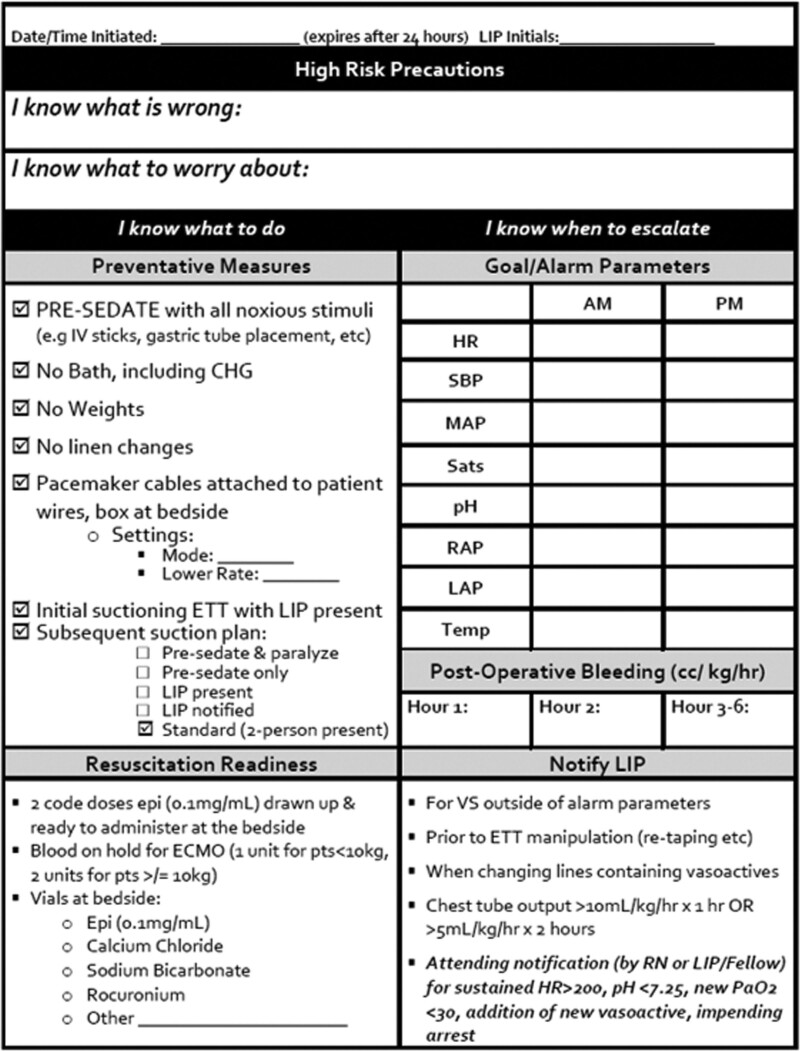
High-risk precautions tool. Bedside tool allows for formal recognition of high-risk patients, limitation of routine care specified alarm parameters, and discussion prompts facilitate the creation of a shared mental model among team members.

The initial intervention was a bedside tool called “High-Risk Precautions” (HRP). This tool emphasized creating a shared mental model, a collective knowledge structure that enabled team members to adapt and respond appropriately in dynamic situations. Additionally, the tool created formal guardrails by articulating patient-specific alarm parameters, resuscitation readiness measures, limitations for noxious stimuli and routine nursing care, and specific criteria for medical team notification (Fig. 1). Finally, incorporating visual aids and structured discussions of at-risk patients into existing workflow heightened overall awareness.

**Fig. 2.**
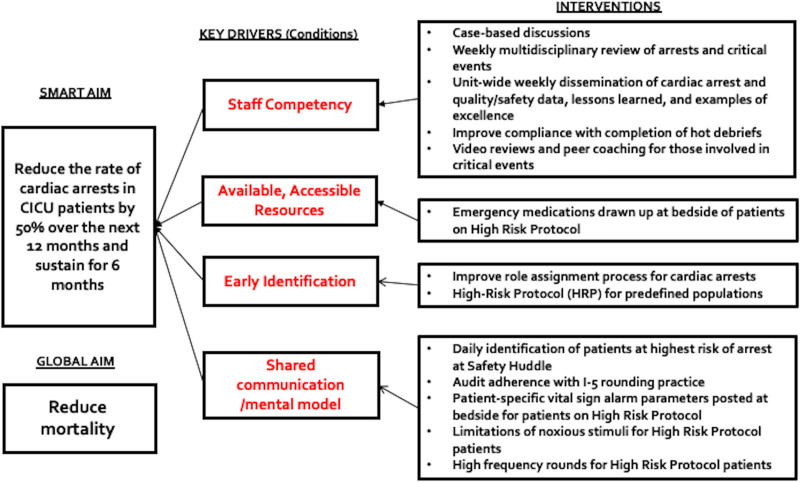
Key driver diagram. Developed by an interdisciplinary workgroup utilizing the Institute for Healthcare Improvement’s model for improvement. Depicts the global and smart aim statements and optimal conditions required to meet them: staff competency, available and accessible human/physical resources, early identification, shared communication, and mental model.

Following the implementation of high-risk precautions, the CA rate fell below baseline for several months. However, these improvements were not sustained, and a multidisciplinary workgroup, including frontline and leadership representation from nursing and medicine and representatives from respiratory care and quality improvement, convened in June 2017 to broaden and reinforce CA reduction efforts. This project was structured using the Institute for Healthcare Improvement’s Model for Improvement.^24,25^ The multidisciplinary workgroup created a key driver diagram (Fig. 2), with the resulting interventions anchored by the high-risk precautions tool embedded within a broader multidisciplinary cardiac arrest reduction program (CARP).

### Statistical Analysis

The baseline CA rate was established by reviewing code sheets, extracting data from the electronic health record, and manually abstracting data for submission to clinical patient registries. The primary outcome measure was adjusted CAs per 1000 CICU patient-days. Multiple CAs in the same patient that occurred within 60 minutes were not considered separate events. We excluded all arrests that occurred during bedside surgical interventions. We used statistical process control methodology to track the change in the CA rate over time, utilizing special cause rules from the Healthcare Data Guide.^26^

We compared the patient population demographics before and after the intervention using Wilcoxon rank-sum tests to analyze the skewed continuous variables and Chi-square tests for the remaining categorical variables. Chi-square tests compared CICU outcomes (CA, CICU major complication, ECPR, and discharge mortality) before and after implementation. A multivariate model adjusted for all unbalanced patient characteristics (*P* < 0.1), using a *P* value <0.05 defining statistical significance. The team conducted all analyses using R version 4.03 (R Foundation for Statistical Computing, 2020; Vienna, Austria).

### Bundle Elements and Project Sustainment

#### High-risk Precautions

Initially, HRP patients included those with specific known risk factors (postoperative patients after stage I single-ventricle palliation or systemic-to-pulmonary artery shunt; premature neonates after intracardiac repair; patients with pulmonary hypertension after bypass; patients in the first 24 hours after surgery with delayed sternal closure) or provider concern. Over time, the workgroup refined these inclusion criteria and the duration of tool utilization. By July of 2018, criteria were aligned with the Pediatric Cardiac Critical Care Consortium (PC^4^) CAP project—a multicenter QI project aimed at arrest reduction based on multicenter data and successful CA reduction efforts at multiple centers, including ours. As outlined by the PC^4^ CAP project, patients assigned high-risk precautions status included neonates after cardiopulmonary bypass surgery, neonates/infants after single-ventricle palliation (pulmonary artery band and systemic-to-pulmonary artery shunt), and medical patients requiring intubation within 4 hours of admission.

Although the application of high-risk precautions was revised since its initial implementation in July 2016, the essential elements remain the same. Guided by the prompts “I Know What is Wrong” and “I Know What to Worry About,” from the I-5 Model for patient handoffs,^27^ the bedside team articulated patient-specific physiologic concerns and vulnerabilities that must be recognized and rapidly addressed. The I-5 Model was designed to validate and verify that each team member had a collective understanding of critical components of patient risk, including patient condition, expected trajectory, and potential threats empathizing a shared mental model among team members.^27^ The care team delineated patient-specific alarm parameters identified and documented noxious stimuli to be avoided entirely (eg, patient bathing on the first postoperative night), or, if unavoidable, done with presedation and provider notification or presence at the bedside (eg, endotracheal tube suctioning). In addition, the protocol specified resuscitation readiness measures, such as blood on hold, extracorporeal membrane oxygenation (ECMO) team awareness, and epinephrine drawn up at the bedside. A packet at each HRP patient bedside summarized these goals, parameters, and guidelines.

#### Weekly Team Meetings

The CARP leadership team held open-door weekly meetings, beginning with an in-depth review of any CAs or near-arrests from the preceding week. Reviews included video and monitor feeds from the patient’s room around the time of the event; relevant documentation (eg, notes or code sheets); details from the post-event hot debrief tool (**see Figure 1, Supplemental Digital Content 1,** http://links.lww.com/PQ9/A357); and feedback from individual provider video reviews. Bedside staff who cared for the patient were strongly encouraged to attend and provide first-person input. Reviews focused on identifying systems and processes that contributed to the arrest event rather than assigning individual failure. In addition, the team evaluated progress on critical interventions and processed metric data weekly.

#### Arrest Debriefs

Each CA was examined through hot and cold debriefs. Hot debriefs occurred immediately after events, assessed individual and team effectiveness, identified systems-level issues, and highlighted examples of high performance. As part of the cold debrief process, video event reviews included staff interviews to improve understanding of cardiac arrest circumstances. In addition, the CARP team reviewed debrief data to identify systems issues and inform Plan-Do-Study-Act (PDSA) cycles.

#### Weekly Arrest Update

Weekly updates via a reporting template included patient diagnosis, cause of arrest (or near arrest), and contributing factors for each event, along with “lessons learned” and praise for specific positive actions. The team emailed this template to all CICU staff after the CARP meeting. The broad dissemination of these findings was essential to ensuring that lessons learned were not limited to the event participants themselves.

#### High-frequency Rounds

The workgroup initiated high-frequency rounds after recognizing that ongoing interdisciplinary discussion is a cornerstone of successful intervention. During a standard day in the CICU, patients are rounded on by the interdisciplinary team twice—with the day and the night team. For high-risk precautions patients, the team rounded two additional times daily. During these focused bedside discussions, team members reviewed the patient’s trajectory since prior bedside rounds and documented it as improving, unchanged, or worsening; solicited new concerns, and implemented new interventions as needed. Furthermore, the bedside nurse documented all concerns and changes at the bedside utilizing a standardized template.

#### Visual Cues

High-risk precautions patients were identified with bright pink “HRP” signs on their doors and highlighted on unit quality dashboards. These visual cues enhanced situational awareness and quickly denoted patient status during daily safety huddles.

## RESULTS

**Fig. 3.**
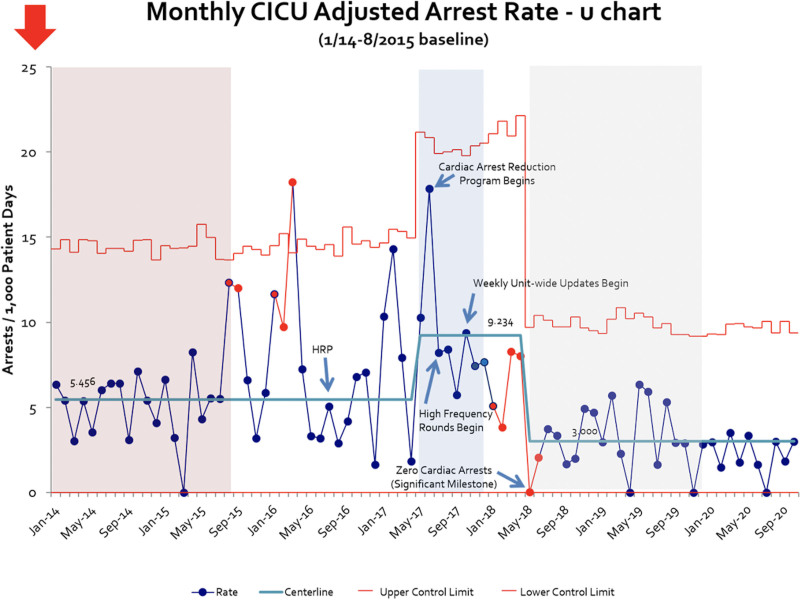
Cardiac arrest control chart. Depicts monthly arrest rate over time and centerline shifts, annotated with pivotal PDSA cycle implementation time points.

Inclusive of the baseline period, there were 3963 admissions during the study period, of which 45.2% were medical and 54.8% surgical. After establishing a baseline arrest rate, we established the CARP workgroup in late June 2017. Change implementation utilizing PDSA cycles began with revision and relaunch of the high-risk precautions tool. It continued until bundle implementation of the six successful cycles described above was completed in November 2017. Figure 3 depicts the control chart annotated with PDSA cycles. Following the program launch, CA rates decreased by 68% compared to baseline and 45% from the historical baseline.

The system remained stable with a centerline of 3.000 adjusted CA per 1000 CICU patient-days after 30 months (May 2018 to October 2020). After launch of the multidisciplinary program, we achieved a significant reduction in the adjusted occurrence of CA (5.25%–4.46%, *P* = 0.022), CICU major complications (17.1%–12.6%, *p < 0*.001), and discharge mortality (5.76%–5.00%, *P* = 0.048; Table 1). There was no increase in balancing measures—ECMO utilization and eCPR incidence—which may have led to increased intensive care unit-related morbidity.

**Table 1.** Adjusted CICU Outcomes Preintervention and Postintervention

Outcome	Pre-CARP January 14–June 17	Post-CARP July 17–October 20	*P*
Admissions	2,074	1,889	
Cardiac arrest	5.25% (4.56%–6.15%)	4.46% (3.93%–5.18%)	0.022
CICU major complication	17.1% (15.4%–18.9%)	12.6% (11.3%–14.1%)	<.001
ECPR	2.00% (1.72%–2.47%)	1.99% (1.71%–2.46%)	0.968
ECMO	2.06% (1.45%–2.87%)	1.92% (1.33%–2.70%)	0.684
Discharge MORTALITY	5.76% (5.06%–6.67%)	5.00% (4.42%–5.77%)	0.048

Adjustment by unbalanced patient demographics (*P* < 0.1).

Logistic regression used to adjust each outcome by age, weight, chromosome abnormality, and STAT category.

Major Complications: cardiac arrest, mechanical circulatory support, bleeding requiring reoperation, unplanned reoperation or reintervention, arrhythmia requiring permanent pacemaker, pleural or pericardial effusion requiring drainage, pulmonary embolism, seizure, IVH > grade II, intracranial hemorrhage, stroke, brain death, paralyzed diaphragm, dialysis or CRRT for acute renal failure, NEC, endocarditis, surgical site infection, UTI, and sepsis.

An analysis of patient demographics and acuity level revealed that the patient population was similar before and after intervention (Table 2). Although there were fewer STAT 5 cases during the improvement era, there was a similar prevalence of single-ventricle physiology and a significantly higher prevalence of chromosomal abnormalities.

**Table 2.** CICU Patient Demographics Population Preintervention and Postintervention

Demographic	Overall	Pre-CARP	Post-CARP	Comparison
	January 14–October 20	January 14–June 17	July 17–October 20	*P*
Admissions	3,963	2,074	1,889	
Admission type				0.469
Medical	1,792 (45.2%)	926 (44.6%)	866 (45.8%)	
Surgical	2,171 (54.8%)	1,148 (55.4%)	1,023 (54.2%)	
Gender				0.810
Male	2,183 (55.1%)	1,147 (55.3%)	1,036 (54.9%)	
Female	1,779 (44.9%)	927 (44.7%)	852 (45.1%)	
Neonate				0.230
Yes	822 (20.7%)	446 (21.5%)	376 (19.9%)	
No	3,141 (79.3%)	1,628 (78.5%)	1,513 (80.1%)	
Age at admission (yrs)	1.33 [0.19–8.29]	1.13 [0.17–7.61]	1.52 [0.23–8.94]	0.012
Weight at admission (kg)	9.10 [4.28–24.9]	8.60 [4.11–24.1]	9.63 [4.40–25.2]	0.061
Prematurity				0.855
Yes	630 (19.5%)	343 (19.4%)	287 (19.7%)	
No	2,597 (80.5%)	1,427 (80.6%)	1,170 (80.3%)	
Chromosomal abnormality				0.003
Yes	853 (21.5%)	408 (19.7%)	445 (23.6%)	
No	3,107 (78.5%)	1,666 (80.3%)	1,441 (76.4%)	
Diagnosis physiology				0.121
Single ventricle	756 (19.1%)	376 (18.1%)	380 (20.1%)	
Biventricular	3,207 (80.9%)	1,698 (81.9%)	1,509 (79.9%)	
STAT category (surg pts only)				0.001
Noncategorizable	41 (1.89%)	17 (1.48%)	24 (2.35%)	
1	760 (35.0%)	360 (31.4%)	400 (39.1%)	
2	674 (31.0%)	380 (33.1%)	294 (28.7%)	
3	234 (10.8%)	135 (11.8%)	99 (9.68%)	
4	386 (17.8%)	207 (18.0%)	179 (17.5%)	
5	76 (3.50%)	49 (4.27%)	27 (2.64%)	

*Admissions with missing or ineligible values are not included:

Gender: 1 (0.03%) missing; Weight: 7 (0.18%) missing; Prematurity: 736 (18.6%) missing (only 9.3% missing for surgical patients); Chromosomal abnormality: 3 (0.08%) missing; and STAT category: 1,792 (45.2%) ineligible.

**Fig. 4.**
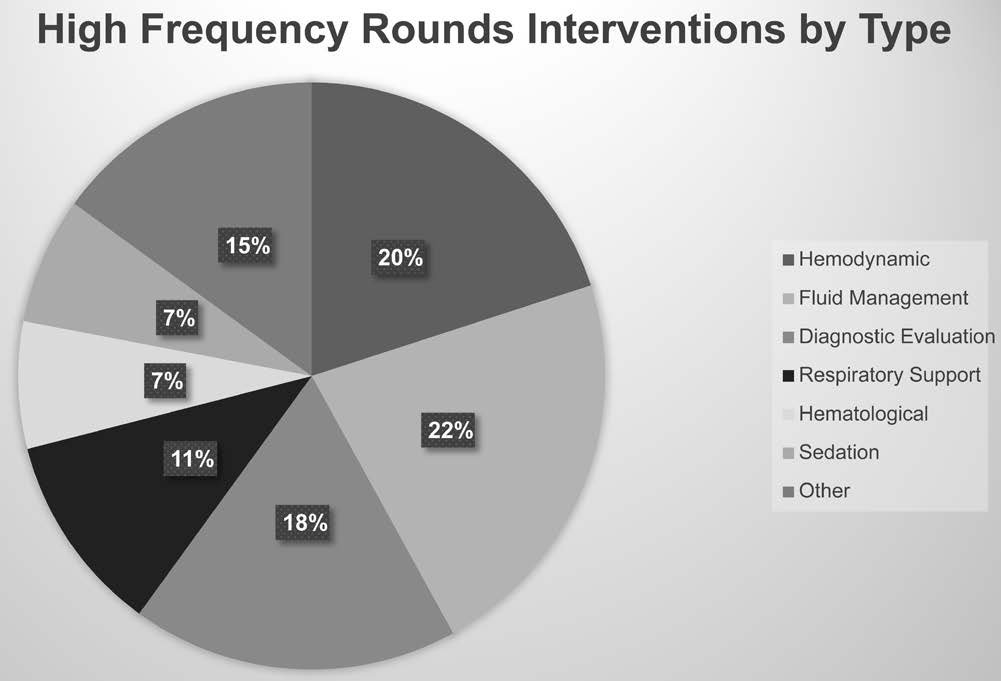
High-frequency rounds documented interventions. Audits of the high-frequency rounding process showed that increasing rounding frequency resulted in modifications to the plan in 76% of patients. Classification of interventions showed that changes in fluid management, hemodynamic management, or diagnostic evaluation were most prevalent.

Audits of the high-frequency rounding process showed that increased rounding frequency resulted in modifications to the plan in 76% of patients. Analysis of the interventions revealed that changes in fluid management (22%), hemodynamic management (20%), or diagnostic evaluation (18%) accounted for the majority of modifications (see Fig. 4 for details).

### Variation Analysis

After reviewing 20 months of CA data preceding the 2015 increase, these months represented special cause variation (outer-third rule), from a baseline of 5456 adjusted CA per 1000 CICU patient-days. During this period, and again during early 2016 when special cause variation was noted (single point outside the control limits), no purposeful systems changes were identified as etiology for this variation, including turnover of surgical staff; however, both periods were notable for CICU attending staff turnover, resulting in a decrease in the average years of experience of the attending staff. These spikes led to heightened awareness of CA, triggering our first intervention to prevent CA, high-risk precautions, in July 2016. Unfortunately, this intervention did not result in a sustained change to the metric.

Signals of concern in the CA rate arose in early 2017, with no apparent etiology identified other than staff turnover. In June 2017, an additional special cause increase in CA was identified (single point outside the control limits). The subsequent six months resulted in another special cause (8 points above the centerline, beginning in May 2017). Based on data from May to December 2017, trial limits produced a new centerline of 9.234 adjusted CA per 1000 CICU patient-days, increasing 69% from the previous center line rate.

Beginning in January 2018, CA data points were consistently below the centerline. Still, as this was in relation to the trial centerline rather than the historical baseline, we elected not to revise the centerline. In May 2018, no CAs occurred, a significant milestone not achieved in over three years. This month also marked the first of 8 consecutive months below the *historical baseline*, suggesting that the implemented changes were likely impacting the system and culture, supporting centerline revision based on this special cause.

## DISCUSSION

After implementing the CARP, the CA arrest rate fell from 9.234 to 3.000 per 1000 CICU patient-days, representing a 68% reduction and a decrease of 45% from the historical baseline of 5456 per 1000 CICU patient-days; this decrease was sustained for 30 months. In addition, there was a significant reduction in unadjusted and adjusted CA rate, major complications, and discharge mortality after intervention implementation. Shifting the paradigm to view CA as a preventable occurrence was an essential first step in this journey. Moreover, conceptualizing CA as a quality metric to be reduced by improvements in the system—rather than a failure attributed to a specific discipline, individual provider, or physiology—has enabled significant gains.

The multidisciplinary engagement was a second and equally critical step toward sustained CA reduction. Clinically and conceptually, the high-risk precautions bedside tool rolled out in 2016 is consistent with the one currently in place; however, the tool did not change the system when released as an isolated effort. Embedding the tool within a multidisciplinary framework identifying bedside providers as critical agents of change, soliciting and incorporating frontline input regularly, and creating a weekly feedback loop of data and lessons learned to providers, created a broad base of engagement fundamental to sustainable gains. For example, Ferguson et al^21^ reported a single targeted intervention, prepared code-dose epinephrine at the bedside of high-risk patients, to decrease CA rates in pediatric cardiac patients from 17.2 per 1000 patient-days to 7.6 per 1000 patient-days.^21^ This was a practice utilized in our initial High-Risk Precautions intervention; however, we did not realize sustained benefit until embedding this initiative in a broader multidisciplinary effort. The ability of bedside teams with a shared mental model to proactively prevent decompensation may explain the enhanced effect of our broader bundle-based initiative.

Multidisciplinary engagement and a unit-wide approach to CA reduction were critical to achieving the culture change we believe is required for significant and sustained CA reduction. The success of this program centers not only on the identification and elevation of patients at high risk of CA but also on the promotion of a culture of self-reflection and interprofessional backing of the bundle. Identifying high-risk patients helps to ensure a shared mental model of the risk matrix both at the bedside and broadly across the unit, and it allows for better matching of resources to risk level. Providing opportunities for staff to review events in both hot and cold debrief forums enhances team performance and allows for individual reflection. Dissemination to all providers in the CICU promotes transparency regarding any systems issues identified, provides positive reinforcement to involved staff, keeps the entire care team aware of unit arrest rates, and shares lessons learned. Sustainability is challenging, and many QI interventions do not show sustained improvement over time. For example, Gaies et al^28^ led a QI collaborative in which 3 of 4 hospitals could not sustain their improvements. The hospital that did sustain improvement attributed its success to the inclusion of multidisciplinary team members, creating a broad culture change, and conducting regular data reviews with transparency on the progress that included the entire team.^28^ Similarly, we believe that these three elements were impactful in our effort to create a sustainable reduction in CA.

This local initiative’s early successes and challenges helped inform the development of a multicenter arrest prevention bundle through PC^4^. It is difficult to quantify how much participation in a multicenter project contributed to the successful sustainment of our early gains. Still, we believe our experience demonstrates the value of a multicenter collaborative learning model that creates a positive feedback loop of local efforts informing multicenter projects, invigorate and support local efforts. Such “all teach, all learn” models facilitate the rapid diffusion of successful single-center efforts and leverage the communication and data-sharing infrastructure of a multicenter collaborative. This model ensures the broader dissemination of best practices, ultimately increasing the opportunity to improve patient outcomes.

There is no control over the natural variation in patient acuity and volume over time; the changes in CA rate may reflect the impact of factors beyond the QI initiatives described here. For example, although there were fewer STAT 5 cases during the improvement era, the prevalence of single-ventricle physiology was similar, and we noted a significantly higher prevalence of chromosomal abnormalities. Both these patient characteristics are known to increase postoperative CA risk.^3^ These differences may reflect an institutional trend toward increased use of the hybrid palliation and do not explain the sustained reduction in CA rate. Moreover, aggregate turnover in nursing, medical, and surgical staff has brought providers with less overall experience into the CICU care team (in addition, all new staff, regardless of prior work experience, have less prior familiarity with this CICU). Thus, the turnover may bias results toward no improvement in CA numbers.^29^ These data represent the experience at a single tertiary care center, and differences in case-mix, unit size, monitoring capabilities, culture, or other characteristics may limit the generalizability of our results. However, by incorporating input from stakeholders across disciplines throughout the process, the QI bundle presented here could be adjusted to align with local resources and practices, facilitating implementation at other centers.

CA occurs more frequently in hospitalized children with cardiovascular disease than in those with any other disease type.^30^ Our efforts are predicated on the assumption that CA is a preventable event. A shared mental model coupled with targeted communication at the unit-wide and bedside levels enables adjustment of resources and monitoring to a patient’s evolving level of risk to proactively prevent arrest. In addition, establishing an interdisciplinary leadership team and deliberative QI process backed by broad stakeholder engagement promoted the culture change required to ultimately assure sustainable gains.

## ACKNOWLEDGMENTS

Amber Merritt, MSN, Monique Powell, RN, Children’s National Hospital Department of Respiratory Care was assisted with the study.

## DISCLOSURE

The authors have no financial interest to declare in relation to the content of this article.

## Supplementary Material


