# Supplementary material for: Shifting the Paradigm: A Quality Improvement Approach to Proactive Cardiac Arrest Reduction in the Pediatric Cardiac Intensive Care Unit
Source: Pediatr Qual Saf. 2022 Jan 21;7(1):e525. doi: 10.1097/pq9.0000000000000525 (PMC8782114; doi:10.1097/pq9.0000000000000525)
Supplement: Supplementary file 1 [file pqs-7-e525-s001.pdf]

Patient Sticker Here

Tool for Event Debrief (TED)

For QI purposes only; privileged and confidential

**Event Date/Time:**	**Location:** CICU HKU	**Type of Event:** CAT / Code Blue / eCPR / Other: ________________
**Huddle Leader:**	**Huddle Start Time:**	**Huddle End Time:**

HUDDLE LEADER

Tell participants about the goals of huddle:

- *Collect **facts** about the event*
- ***This is not a test** –no "right answers"*
- *Gain insight into how our team works*
- ***complete small forms***
- *Should take **5 minutes or less***
- *Try to complete as close to event as possible*

PLEASE COMPLETE THE FOLLOWING

- ☐ TED tool (this packet)
- ☐ Participant feedback cards (collect all)
- ☐ If applicable: telemetry *(print or copy EKG)*
- ☐ Yellow copy of code recording form
- ☐ Put packet in BEAR basket
- ☐ Email notification of event to BEARdebrief@childrensnational.org

TIPS

Focus Participants on:

- Their actions/thoughts rather than those of others
- Direct and respectful words

Address emotional responses *following* fact collection when possible

Need help facilitating? Email us!
BEARDebrief@childrensnational.org

*Discuss the below questions (this column only) with the whole group **after small forms are completed.***

Systems- Level Issues:

Items for follow-up:

Lessons Learned/What went well?:

End result of event (patient disposition):

1. **Was this patient identified as high risk (watcher, "hot spot," etc)?**
   Yes /No
   a. If yes, to what degree was decompensation anticipated?
      Strongly / Somewhat / Neutral / Not at all
      Details:
2. **What was the patient : nurse assignment for this patient?**
   >3:1   3:1   2:1   1:1   1:>1
3. **Were roles assigned?** No/Yes (by whom?)
   ____
4. **Briefly describe:**
   - ☐ Problems with equipment/supplies:
   - ☐ Other concurrent events in the unit:
5. **The following roles were assigned (names):**

	MD Team Lead	
	RN Team Lead	
	Pt's RN /RN 1	
	Recorder	
	Med prep	
	CPR	
	Respiratory	
	"Runner"	
	Other	
	Other:	
	RN 2 (if applicable)	
	MD Assist (if applicable)	

Briefly describe the patient(history, reason for admission):

*Please place in BEAR bins in CICU/HKU when completed **and** send an email to BEARdebrief@childrensnational.org*

My role(s) in THIS event (ex: recorder, CPR, etc) ______________________________________

What caused this patient's clinical worsening?

What was the first action YOU took to help address the problem? (not including escalation/ calling for help)

What interventions were completed/considered to treat the underlying problem?

How well did I as an individual perform? 1 2 3 4 5 (1-poorly; 3-well; 5–perfectly)

How well did we perform as a team? 1 2 3 4 5 (1-poorly; 3-well; 5–perfectly)

How well did the systems around us function? 1 2 3 4 5 (1-poorly; 3-well; 5–perfectly)

How well did we provide high quality, safe care? 1 2 3 4 5 (1-poorly; 3-well; 5–perfectly)

Did any team member perform exceptionally well? (name) ______________________________

Was your role in this event assigned or assumed?

How frequently have you performed this role before this event?
Never / rarely / sometimes / often / very often

How did you know what tasks to complete during this event?

What Went Well	What Could Be Better

My role(s) in THIS event (ex: recorder, CPR, etc) ______________________________________

What caused this patient's clinical worsening?

What was the first action YOU took to help address the problem? (not including escalation/ calling for help)

What interventions were completed/considered to treat the underlying problem?

How well did I as an individual perform? 1 2 3 4 5 (1-poorly; 3-well; 5–perfectly)

How well did we perform as a team? 1 2 3 4 5 (1-poorly; 3-well; 5–perfectly)

How well did the systems around us function? 1 2 3 4 5 (1-poorly; 3-well; 5–perfectly)

How well did we provide high quality, safe care? 1 2 3 4 5 (1-poorly; 3-well; 5–perfectly)

Did any team member perform exceptionally well? (name) ______________________________

Was your role in this event assigned or assumed?

How frequently have you performed this role before this event?
Never / rarely / sometimes / often / very often

How did you know what tasks to complete during this event?

What Went Well	What Could Be Better

Please place in BEAR bins in CICU/HKU when completed ***and*** *send an email to BEARdebrief@childrensnational.org*

My role(s) in THIS event (ex: recorder, CPR, etc) ____

What caused this patient's clinical worsening?

What was the first action YOU took to help address the problem? (not including escalation/ calling for help)

What interventions were completed/considered to treat the underlying problem?

How well did I as an individual perform? 1 2 3 4 5 (1-poorly; 3-well; 5–perfectly)

How well did we perform as a team? 1 2 3 4 5 (1-poorly; 3-well; 5–perfectly)

How well did the systems around us function? 1 2 3 4 5 (1-poorly; 3-well; 5–perfectly)

How well did we provide high quality, safe care? 1 2 3 4 5 (1-poorly; 3-well; 5–perfectly)

Did any team member perform exceptionally well? (name) ________________________________

Was your role in this event assigned or assumed?

How frequently have you performed this role before this event?
Never / rarely / sometimes / often / very often

How did you know what tasks to complete during this event?

What Went Well	What Could Be Better

My role(s) in THIS event (ex: recorder, CPR, etc) ____

What caused this patient's clinical worsening?

What was the first action YOU took to help address the problem? (not including escalation/ calling for help)

What interventions were completed/considered to treat the underlying problem?

How well did I as an individual perform? 1 2 3 4 5 (1-poorly; 3-well; 5–perfectly)

How well did we perform as a team? 1 2 3 4 5 (1-poorly; 3-well; 5–perfectly)

How well did the systems around us function? 1 2 3 4 5 (1-poorly; 3-well; 5–perfectly)

How well did we provide high quality, safe care? 1 2 3 4 5 (1-poorly; 3-well; 5–perfectly)

Did any team member perform exceptionally well? (name) ________________________________

Was your role in this event assigned or assumed?

How frequently have you performed this role before this event?
Never / rarely / sometimes / often / very often

How did you know what tasks to complete during this event?

What Went Well	What Could Be Better

*Please place in BEAR bins in CICU/HKU when completed **and** send an email to BEARdebrief@childrensnational.org*

Tool for Event Debrief (TED)

For QI purposes only: privileged and confidential

My role(s) in THIS event (ex: recorder, CPR, etc) ____

What caused this patient's clinical worsening?

What was the first action YOU took to help address the problem? (not including escalation/ calling for help)

What interventions were completed/considered to treat the underlying problem?

How well did I as an individual perform? 1 2 3 4 5 (1-poorly; 3-well; 5 –perfectly)

How well did we perform as a team? 1 2 3 4 5 (1-poorly; 3-well; 5 –perfectly)

How well did the systems around us function? 1 2 3 4 5 (1-poorly; 3-well; 5 –perfectly)

How well did we provide high quality, safe care? 1 2 3 4 5 (1-poorly; 3-well; 5 –perfectly)

Did any team member perform exceptionally well? (name) ________________________________

Was your role in this event assigned or assumed?

How frequently have you performed this role before this event?
Never / rarely / sometimes / often / very often

How did you know what tasks to complete during this event?

What Went Well	What Could Be Better

My role(s) in THIS event (ex: recorder, CPR, etc) ____

What caused this patient's clinical worsening?

What was the first action YOU took to help address the problem? (not including escalation/ calling for help)

What interventions were completed/considered to treat the underlying problem?

How well did I as an individual perform? 1 2 3 4 5 (1-poorly; 3-well; 5 –perfectly)

How well did we perform as a team? 1 2 3 4 5 (1-poorly; 3-well; 5 –perfectly)

How well did the systems around us function? 1 2 3 4 5 (1-poorly; 3-well; 5 –perfectly)

How well did we provide high quality, safe care? 1 2 3 4 5 (1-poorly; 3-well; 5 –perfectly)

Did any team member perform exceptionally well? (name) ________________________________

Was your role in this event assigned or assumed?

How frequently have you performed this role before this event?
Never / rarely / sometimes / often / very often

How did you know what tasks to complete during this event?

What Went Well	What Could Be Better

*Please place in BEAR bins in CICU/HKU when completed **and** send an email to BEARdebrief@childrensnational.org*

Tool for Event Debrief (TED)

For QI purposes only; privileged and confidential

My role(s) in THIS event (ex: recorder, CPR, etc) ____________________________________

What caused this patient's clinical worsening?

What was the first action YOU took to help address the problem? (not including escalation/ calling for help)

What interventions were completed/considered to treat the underlying problem?

How well did I as an individual perform? 1 2 3 4 5 (1-poorly; 3-well; 5–perfectly)

How well did we perform as a team? 1 2 3 4 5 (1-poorly; 3-well; 5–perfectly)

How well did the systems around us function? 1 2 3 4 5 (1-poorly; 3-well; 5–perfectly)

How well did we provide high quality, safe care? 1 2 3 4 5 (1-poorly; 3-well; 5–perfectly)

Did any team member perform exceptionally well? (name) ____________________________

Was your role in this event assigned or assumed?

How frequently have you performed this role before this event?
Never / rarely / sometimes / often / very often

How did you know what tasks to complete during this event?

What Went Well	What Could Be Better

My role(s) in THIS event (ex: recorder, CPR, etc) ____________________________________

What caused this patient's clinical worsening?

What was the first action YOU took to help address the problem? (not including escalation/ calling for help)

What interventions were completed/considered to treat the underlying problem?

How well did I as an individual perform? 1 2 3 4 5 (1-poorly; 3-well; 5–perfectly)

How well did we perform as a team? 1 2 3 4 5 (1-poorly; 3-well; 5–perfectly)

How well did the systems around us function? 1 2 3 4 5 (1-poorly; 3-well; 5–perfectly)

How well did we provide high quality, safe care? 1 2 3 4 5 (1-poorly; 3-well; 5–perfectly)

Did any team member perform exceptionally well? (name) ____________________________

Was your role in this event assigned or assumed?

How frequently have you performed this role before this event?
Never / rarely / sometimes / often / very often

How did you know what tasks to complete during this event?

What Went Well	What Could Be Better

*Please place in BEAR bins in CICU/HKU when completed **and** send an email to BEARdebrief@childrensnational.org*

My role(s) in THIS event (ex: recorder, CPR, etc) ______________________________________

What caused this patient's clinical worsening?

What was the first action YOU took to help address the problem? (not including escalation/ calling for help)

What interventions were completed/considered to treat the underlying problem?

How well did I as an individual perform? 1 2 3 4 5 (1-poorly; 3-well; 5–perfectly)

How well did we perform as a team? 1 2 3 4 5 (1-poorly; 3-well; 5–perfectly)

How well did the systems around us function? 1 2 3 4 5 (1-poorly; 3-well; 5–perfectly)

How well did we provide high quality, safe care? 1 2 3 4 5 (1-poorly; 3-well; 5–perfectly)

Did any team member perform exceptionally well? (name) ______________________________

Was your role in this event assigned or assumed?

How frequently have you performed this role before this event?
Never / rarely / sometimes / often / very often

How did you know what tasks to complete during this event?

What Went Well	What Could Be Better

My role(s) in THIS event (ex: recorder, CPR, etc) ______________________________________

What caused this patient's clinical worsening?

What was the first action YOU took to help address the problem? (not including escalation/ calling for help)

What interventions were completed/considered to treat the underlying problem?

How well did I as an individual perform? 1 2 3 4 5 (1-poorly; 3-well; 5–perfectly)

How well did we perform as a team? 1 2 3 4 5 (1-poorly; 3-well; 5–perfectly)

How well did the systems around us function? 1 2 3 4 5 (1-poorly; 3-well; 5–perfectly)

How well did we provide high quality, safe care? 1 2 3 4 5 (1-poorly; 3-well; 5–perfectly)

Did any team member perform exceptionally well? (name) ______________________________

Was your role in this event assigned or assumed?

How frequently have you performed this role before this event?
Never / rarely / sometimes / often / very often

How did you know what tasks to complete during this event?

What Went Well	What Could Be Better

*Please place in BEAR bins in CICU/HKU when completed **and** send an email to BEARdebrief@childrensnational.org*

My role(s) in THIS event (ex: recorder, CPR, etc) ______________________________________

What caused this patient's clinical worsening?

What was the first action YOU took to help address the problem? (not including escalation/ calling for help)

What interventions were completed/considered to treat the underlying problem?

How well did I as an individual perform? 1 2 3 4 5 (1-poorly; 3-well; 5–perfectly)

How well did we perform as a team? 1 2 3 4 5 (1-poorly; 3-well; 5–perfectly)

How well did the systems around us function? 1 2 3 4 5 (1-poorly; 3-well; 5–perfectly)

How well did we provide high quality, safe care? 1 2 3 4 5 (1-poorly; 3-well; 5–perfectly)

Did any team member perform exceptionally well? (name) ______________________________

Was your role in this event assigned or assumed?

How frequently have you performed this role before this event?
Never / rarely / sometimes / often / very often

How did you know what tasks to complete during this event?

What Went Well	What Could Be Better

My role(s) in THIS event (ex: recorder, CPR, etc) ______________________________________

What caused this patient's clinical worsening?

What was the first action YOU took to help address the problem? (not including escalation/ calling for help)

What interventions were completed/considered to treat the underlying problem?

How well did I as an individual perform? 1 2 3 4 5 (1-poorly; 3-well; 5–perfectly)

How well did we perform as a team? 1 2 3 4 5 (1-poorly; 3-well; 5–perfectly)

How well did the systems around us function? 1 2 3 4 5 (1-poorly; 3-well; 5–perfectly)

How well did we provide high quality, safe care? 1 2 3 4 5 (1-poorly; 3-well; 5–perfectly)

Did any team member perform exceptionally well? (name) ______________________________

Was your role in this event assigned or assumed?

How frequently have you performed this role before this event?
Never / rarely / sometimes / often / very often

How did you know what tasks to complete during this event?

What Went Well	What Could Be Better

*Please place in BEAR bins in CICU/HKU when completed **and** send an email to BEARdebrief@childrensnational.org*
